# High preterm delivery rates associated with initiation of HAART during pregnancy

**DOI:** 10.1186/1758-2652-13-S4-P158

**Published:** 2010-11-08

**Authors:** GP Taylor, M Douglas, J Smith

**Affiliations:** 1 Imperial College London, Infectious Diseases, London, UK; 2 Imperial College Healthcare NHS Trust, Obstetrics & Gynaecology, London, UK

## Purpose of the study

To understand the relationship between HAART and preterm delivery (PTD).

## Methods

Analysis of prospectively collected data from all patients attending HIV antenatal services at a single UK centre 1995—2010. Fisher's exact test two tailed for categorical variables. T-test for continuous variables.

## Results

Data are presented on all 324 deliveries up to 26/07/2010. 78% of the women were Black African, 10.3% Caucasian. Risk for HIV infection was heterosexual intercourse in 94%, injecting drug use in 1.2%. Median gestational age at first ante-natal clinic appointment was 13 weeks. 7 women took no therapy prior to delivery and 7 took dual NRTI treatment. Data on the remainder are summarised in Table 1

**Table 1:** 

Treatment	Number	Median CD4 /μL (Baseline ANC)	Median HIV viral load/ml	PTD Number (%)
Zidovudine monotherapy	61	445	2513	4 (6.6%)

START	64	360	8930	16 (25%)

new continuous HAART	58	150	23430	10 (17.2%)

pre-conception HAART	127	385	49	16 (12.6%)

Delivery before 37 weeks (PTD) occurred in 14.4% pregnancies of which 62% (8.8% of all pregnancies) before 34 weeks. Difference not significant comparing PTD in patients starting new continuous HAART (17.2%) with pre-conception HAART (12.6%). PTD occurred in 17/142 (12.%) women treated with nevirapine-based HAART compared with 24/102 (23.5%) treated with protease inhibitor (PI)-based HAART (p 0.02). Of the 64 patients treated with a short-course of HAART (START) during pregnancy to prevent mother-to-child HIV transmission 33 were eligible, if willing to delivery by pre-labour caesarean section (PLCS) to receive zidovudine monotherapy (ZDVm) according to the 2008 BHIVA guidelines^1^. Of these 10 had a preterm delivery (30.3%).

## Conclusions

The role of HAART and PI-based HAART in PTD has been controversial. Even in this single-centre study, where all women were managed by one team in accordance with national guidelines, confounders abound. CD4 counts and viral loads differed significantly between patients starting HAART in pregnancy and those taking ZDVm or already on HAART at conception. We therefore compared PTD rates in women who were eligible for, and chose between, START (and the potential of a normal vaginal delivery) and ZDVm with PLCS. The rates of PTD were significantly higher (p 0.005)with START (30.3%) than with ZDVm (6.6%) in these ZDVm eligible mothers. These data suggest that PI-based HAART initiated during pregnancy is associated with a significantly increased rate of PTD and that this is influenced by maternal immune status. Further investigation to determine whether other regimens may have less impact on PTD are urgently required particularly with the increasing use of HAART in prevention of mother-to-child transmission.
